# To be or not to be a protein coding mutation, that’s the question!

**DOI:** 10.1093/nargab/lqaf168

**Published:** 2025-11-21

**Authors:** Dylan De Groote, Daniele Pepe, Xander Janssens, Kim De Keersmaecker

**Affiliations:** Laboratory for Disease Mechanisms in Cancer, Department of Oncology, KU Leuven, Leuven 3000, Belgium; Laboratory for Disease Mechanisms in Cancer, Leuven Cancer Institute, Leuven 3000, Belgium; Laboratory for Disease Mechanisms in Cancer, Department of Oncology, KU Leuven, Leuven 3000, Belgium; Laboratory for Disease Mechanisms in Cancer, Leuven Cancer Institute, Leuven 3000, Belgium; Laboratory for Disease Mechanisms in Cancer, Department of Oncology, KU Leuven, Leuven 3000, Belgium; Laboratory for Disease Mechanisms in Cancer, Leuven Cancer Institute, Leuven 3000, Belgium; Laboratory for Disease Mechanisms in Cancer, Department of Oncology, KU Leuven, Leuven 3000, Belgium; Laboratory for Disease Mechanisms in Cancer, Leuven Cancer Institute, Leuven 3000, Belgium

## Abstract

Accurate annotation of genetic variants—distinguishing whether they affect protein-coding or noncoding genomic regions—is crucial for evaluating their potential role in disease development. Prominent examples have been identified of variants that for many years had been considered to be coding missense or synonymous mutations targeting one gene, and that recently turned out to be noncoding variants, sometimes even modulating a shared regulatory region of multiple genes. These errors were caused by annotating to a canonical reference transcript, whereas an alternative transcript was in reality expressed in respect to which the mutations have a different annotation. Unfortunately, this practice of annotating genetic variants to a reference transcript, without verifying whether this transcript is expressed or whether the mutation causes a change of expressed transcript, is still widespread. However, the implementation of RNA sequencing and availability of these data in online portals allow to verify expressed transcripts in relevant tissues. Integration of DNA- and RNA-sequencing data, in which detected DNA mutations are annotated in respect to the transcripts that are expressed in the corresponding tissue or disease sample as detected by RNA sequencing, avoids misinterpretation of noncoding variants as coding and *vice versa*, thereby improving the functional interpretation of genetic variants.

## Graphical Abstract

**Graphical Abstract:**
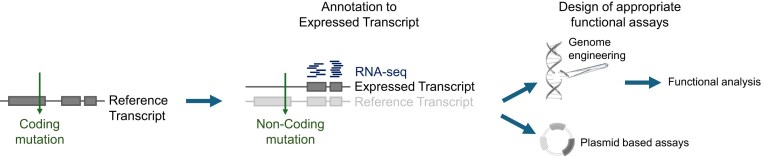

## Introduction

Genetic variants or mutations, whether congenital or somatically acquired, form the basis of many human diseases. These mutations can include large structural aberrations such as chromosomal translocations or large copy number variations, insertions or deletions of a limited number of nucleotides (INDELs), or single nucleotide variants (SNVs). SNVs are the most common type of mutations and are classified as coding or noncoding mutations based on their location in the genome. Coding mutations occur within the protein-coding sequences of genes, and can be missense (amino acid substitution), synonymous (silent, no amino acid change), nonsense (introducing a premature stop codon), or nonstop (carboxy-terminal protein extension) [1–3]. SNVs can alter the resulting protein’s structure, stability, or function. This potentially leads to loss-of-function or gain-of-function phenotypes, depending on the gene and the nature of the mutation. A nice illustration of this is for instance the p.Gly12Asp missense mutation in the *KRAS* gene, which is frequently detected in a variety of cancer types. KRAS is a GTPase that functions as a molecular switch, cycling between an inactive GDP-bound state and an active GTP-bound state. In this function, it plays a critical role in signal transduction pathways that regulate cell proliferation and survival. The KRAS p.Gly12Asp substitution impairs both intrinsic and GTPase-activating protein-mediated hydrolysis of GTP. This leads to the sustained accumulation of KRAS in its active, GTP-bound form. Consequently, this promotes uncontrolled cellular proliferation and survival, contributing to carcinogenesis [4].

Noncoding mutations occur outside of protein-coding regions. They can target gene regulatory elements such as promoters, enhancers, silencers, or insulators, as well as microRNA (miRNA) binding sites or splicing regulatory regions. While these mutations do not alter the protein sequence directly, they can have profound consequences for gene expression regulation. For example, a mutation in a promoter or enhancer element can disrupt or enhance transcription factor binding, thereby affecting transcriptional initiation rates [5, 6]. A well-established example is the mutations in the promoter of the telomerase reverse transcriptase (*TERT*) gene in glioblastoma brain and melanoma skin tumors. These mutations induce TERT expression by creating a GABP transcription factor binding site [7–10].

Thus, both coding and noncoding mutations can significantly impact gene function and hence can play a role in disease pathobiology. However, the mechanisms by which they perturb gene function, either by modifying the protein product itself or by perturbing its expression dynamics, and the resulting cell biological consequences, can differ greatly. Interestingly, the annotation of certain mutations has changed over time. Indeed, several examples have now been established of mutations that were initially considered to be coding, but that were afterward reannotated as noncoding mutations. This will be the topic of this perspective, in which we will explain the causes of such initial mutational misannotation. Furthermore, we will elaborate on how such misannotations can be avoided and discuss remaining challenges in mutational annotation.

This perspective focuses on variant misannotation related to erroneous classification of mutations as coding or noncoding. Several other types of variant annotation errors exist, such as pathogenic misclassification, which refers to incorrect labeling of mutations as pathogenic or benign [11–13]. While correct coding/noncoding annotation of mutations is an important step toward correct interpretation of the pathogenic role of mutations, pathogenic misclassification is not the main subject of this perspective.

## Reannotation of coding variants to noncoding gene promoter mutations

A first type of mutational misannotation that we would like to highlight is a number of cancer mutations that were initially described as missense and synonymous and that were subsequently reannotated as noncoding gene promoter mutations. In this first class of misannotation examples, the mutations themselves do not influence the coding/noncoding nature of the mutation, and the misannotation stems from mutational annotation in respect to a canonical reference transcript that is irrelevant in the affected tissue because it is not expressed.

**Figure 1.**
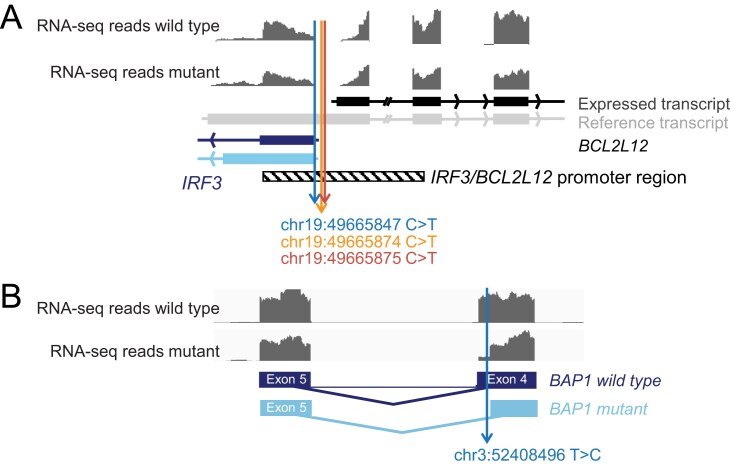
Reannotation of coding to noncoding mutations based on expressed isoform analysis. (**A**) RNA-seq data from melanoma tumors were mapped against hg38 in Integrative Genomics Viewer (IGV). A representative RNA-seq read distribution plot of a wild type and a mutant tumor is shown. The genomic location of the *IRF3/BCL2L12* promoter variants is indicated, as well as the relevant *IRF3* and *BCL2L12* transcripts. (**B**) IGV mapping of RNA-seq data from a representative *BAP1* wild type and mutated tumor. The genomic location of the *BAP1* variants is indicated, as well as wild-type BAP1 transcript and the splice variant generated by the mutant. RNA-seq BAM files of tumors shown in this figure were downloaded from NCI Genomics Data Commons after having obtained dbGaP Authorized Access to dataset phs003014 [NCI’s Datasets for General Research Use, study accession phs000178.v11.p8—The Cancer Genome Atlas (TCGA)].

A first example illustrating this concept is the chr19:49665874 C > T (GRCh38/hg38) mutation, which was described in 2013 as a synonymous p.Phe17Phe mutation targeting the *BCL2L12* oncogene in 4% of melanoma skin cancers [14]. The experimental work that was conducted to study this mutation in 2013 supported the idea that this “synonymous” mutation impairs the binding of miRNA hsa-miR-671–5p to the *BCL2L12* messenger RNA (mRNA), leading to BCL2L12 overexpression and cellular resistance to UV-induced apoptosis. *BCL2L12* is however a gene for which Ensembl reports 19 different transcripts (GRCh38.p13), and NCBI and UCSC (GRCh38/hg38) contain 14 *BCL2L12* transcripts. Depending on which of these transcripts is expressed, the chr19: 49665874 C > T variant corresponds to either a synonymous *BCL2L12* mutation, a *BCL2L12* 5′UTR mutation, or an upstream noncoding mutation. Analysis of RNA-sequencing (RNA-seq) data from human melanoma samples revealed that this variant is an upstream noncoding *BCL2L12* variant in respect to the *BCL2L12* transcripts that are expressed. Even more interestingly, these analyses revealed that this noncoding variant is part of a cluster of noncoding mutations that target the shared promoter region of the *IRF3* and *BCL2L12* genes that run in opposite directions (Fig. 1A). DNase-seq data from primary melanocytes support that the wild-type *IRF3*/*BCL2L12* promoter region corresponds to an open, transcriptionally active chromatin state, and transcription factor binding prediction tools consistently predict that the *IRF3/BCL2L12* promoter mutations disrupt binding motifs for multiple ETS family transcription factors. The functionality of these noncoding mutations is further supported by the fact that they cause downregulation of *IRF3, BCL2L12*, and *TP53* and its target gene *CDKN1A* (*p21*) in CRISPR–Cas9 primary melanocyte models and in mutated human melanoma tumor samples. Interestingly, these noncoding mutations also tend to associate with a worse response to immunotherapy in melanoma patients, which may relate to the function of IRF3 (interferon regulatory factor 3) in innate immunity [15].

A second example of coding to noncoding reannotation is the chr15:40382906 C > T mutation. This variant was first reported in 2014 as a missense p.Ser24Phe substitution in KNSTRN and was described in 19% of cutaneous squamous cell carcinomas (SCCs), a less aggressive skin cancer subtype derived from keratinocytes, as well as in 5% of melanoma tumors [16]. Functional studies in which wild-type or p.Ser24Phe mutant KNSTRN complementary DNA (cDNA) were expressed in keratinocytes, as well as analyses on wild-type versus mutant SCC samples, supported that the identified *KNSTRN* mutation compromises accurate chromosome segregation during cell replication, contributing to aneuploidy and enhanced oncogene-driven tumor growth *in vivo*.

However, also the interpretation of this variant is complicated by 16 different *KNSTRN* transcripts that are reported in Ensembl. Whereas chr15:40382906 C > T indeed corresponds to a KNSTRN p.Ser24Phe mutation in respect to reference transcript *KNSTRN-201*, analysis of RNA-seq data from human melanoma tumors revealed that this transcript is not expressed in melanoma. Intriguingly, chr15:40382906 C > T resides upstream of the expressed *KNSTRN* transcripts in melanoma, and it is part of a cluster of noncoding mutations in the *KNSTRN* promoter region. Transcription factor binding prediction tools suggested only minor effects of the chr15:40382906 C > T variant on transcription factor binding, and it does not significantly impact promoter activity in luciferase reporter assays. However, another variant in the cluster of *KNSTNR* promoter mutations, chr15:40382931G > A, induced a 60% reduction in reporter activity in this assay. Epigenomic profiling by DNase-seq indicates that the genomic region harboring this variant exhibits characteristics of active regulatory elements in melanocytes, marked by open chromatin and H3K27ac and H3K4me3 histone modifications [15].

The examples above illustrate that mutational coding/noncoding misannotation is a recurrent problem, caused by annotating mutations to a canonical reference transcript. Such reference transcripts are not defined based on expression in (a specific) tissue but are identified using the APPRIS (Annotating Principal And Alternatively spliced Isoforms) system. APPRIS uses computational methods like conservation, expression, and protein length to identify the most functionally important transcript for a gene [17]. As the examples above illustrate, such a reference transcript can be different from what is truly expressed in human tissues. The resulting misannotation that can arise when only considering reference transcripts for mutational annotation causes an underestimation of the number of (functionally relevant) noncoding mutations. In fact, recent work from our team suggests that up to 22% of tested mutations (11/50) that are annotated as coding missense or synonymous in melanoma are in reality noncoding promoter mutations [15], illustrating the need for researchers to be aware of the importance of verifying expressed transcripts when annotating mutations.

## Reannotation of coding to intronic splice site mutations

In addition to the examples above where the mutations themselves do not influence the expressed transcripts and hence the mutational annotation, a second class of mutations exists that does affect the expressed transcript isoforms. In this regard, splice site mutations are of high interest: whereas the wild-type allele can be in a coding region, resulting in a synonymous or missense variant call when a mutation is detected, the mutated allele can cause expression of an alternatively spliced transcript. In respect to this alternative splice variant, the mutant allele can be intronic noncoding.

The work by Jayasinghe *et al.* nicely illustrates this concept [18]. In this study, a comprehensive analysis was performed across 8656 tumor samples representing 33 cancer types, integrating DNA mutation calls with corresponding RNA–seq data available in TCGA to identify splice–site–creating mutations (SCMs). A bioinformatic tool, MiSplice, was applied to systematically detect novel splice junctions generated by mutations, followed by manual curation of all SCMs. Case–control comparisons were conducted within each cancer type, comparing isoform expression in each mutant sample to controls lacking the mutation in the same gene. After manual curation, 1964 SCMs were identified. Whereas 52% (1016) of these were in previously annotated splice sites, 37% (721) of the SCMs had been misannotated before as missense and synonymous mutations [18].

A first concrete example identified in this study and illustrating the reannotation of a coding to an intronic mutation is the chr3: 52408496 T > C variant in *BAP1* in kidney renal clear cell carcinoma. This variant is conventionally annotated as a missense p.Asn78Ser mutation. Evaluation of RNA-seq data from mutated versus wild-type patients revealed that this variant creates a *de novo* splice donor site, resulting in expression of an alternative transcript in respect to which the variant is intronic (Fig. 1B). This alternative splicing event results in an in-frame deletion of nine amino acids. When considering total transcript levels, *BAP1* expression levels of mutant versus control samples were relatively comparable, but at protein level, expression of the mutant was significantly lower, suggesting that this conventionally annotated missense mutation in *BAP1* creates an alternatively, intronically spliced transcript that is not readily expressed at protein level [18].

Interestingly, this type of mutational misannotation extends beyond somatic cancer mutations and is also highly relevant in the context of genetic disease syndromes. For example, in Alport syndrome, a hereditary kidney disorder, where a previously reported synonymous variant located in exon 13 of *COL4A3* (p.Thr255Thr) was recently shown to alter splicing and induce skipping of exon 13, resulting in a truncated COL4A3 protein product [19]. In epilepsy, mutations in the *SCN1A* gene have been described. Sparber *et al.* investigated the impact of 21 exonic *SCN1A* variants on *SCN1A* pre-mRNA splicing using splicing reporter assays [20]. Their study revealed that splicing-disruptive variants in *SCN1A* are substantially misannotated, and that 15 of the analyzed exonic variants (71%)—initially classified as “missense,” “synonymous,” “nonsense,” or “in-frame deletions”—were affecting splicing, as also predicted by the bioinformatic tool SpliceAI. Of particular interest is the *SCN1A* variant chr2:166043691 A > G, formerly annotated as a missense p.Asp674Gly mutation, which turned out to be an intronic variant in respect to the expressed transcript. Altogether, these examples again illustrate that careful integration of DNA- and RNA-seq data are vital for correctly interpreting mutational consequences.

## Reannotation of noncoding to coding variants

In the examples above, we have mainly highlighted the reannotation of coding missense/synonymous mutations toward noncoding mutations based on expressed transcripts. Conversely, mutations that were previously thought to be noncoding may, in theory, also correspond to coding mutations in respect to expressed transcripts. Whereas fewer examples are available of this concept, potentially due to lower interest in noncoding mutations, noncoding mutations have been identified that affect splicing and cause expression of a novel transcript in respect to which the mutation is coding. An example is an intronic *STK11* chr19:1221013 G > A mutation that was identified in head and neck cancer. This mutation generates a novel splice site, resulting in inclusion of a novel exon of 130 bp in the STK11 transcript where this mutation is located, thus resulting in a coding mutation in respect to this novel expressed mutated transcript. Inclusion of these extra 130 bp in the transcript causes a frameshift, and the mutation thereby causes a loss of function [21, 22].

Because of the recent development and more widespread implementation of long-read RNA-seq technology, we can expect that more examples of reannotation of noncoding to coding variants will be identified. Long-read RNA-seq technology allows detection of a larger diversity of expressed transcripts in various tissues compared with traditional reference transcripts described in GENCODE. As a consequence, a detected variant can be located in an open reading frame of a tissue-specific novel transcript, and can as such become coding [23]. In this regard, we would also like to mention the GENCODE Capture Long-read Sequencing (CLS) project, a component of the GENCODE project that aims to improve gene annotation of human and mouse genomes. By designing specific capture arrays to target genomic regions and performing long-read sequencing, CLS helps to accurately define transcript structures for long noncoding RNAs and other genes, providing a richer and more precise reference for the research community [24]. Also, in GTEx, a database reporting expressed transcripts in a variety of healthy human tissues, long-read RNA-seq datasets from different tissues are available [23].

## Improved mutation annotation by evaluating expressed transcripts

The general practice of annotating mutations in respect to a reference transcript is unfortunately still very widespread. For instance, cancer genomics databases that are utilized by the entire cancer research community such as the Catalogue of Somatic Mutations in Cancer (COSMIC) and the Genomic Data Commons by default report mutations annotated in respect to a reference transcript, resulting in the mutation annotation errors as described above. Also, in the field of genetic diagnosis of rare diseases, this mutation annotation problem has been recognized [25]. In COSMIC, researchers have the option to select other transcripts to which mutations should be annotated, but the database does not allow to verify the expressed transcripts in relevant tumor samples. Nevertheless, researchers nowadays have a variety of options to verify which transcripts are expressed. For instance, the GTEx portal allows users to easily verify expressed isoforms in healthy human tissues [26]. For this purpose, there is the GTEx “Transcript Browser”(https://www.gtexportal.org/home/transcriptPage), where users can enter a gene name and verify isoform expression, exon expression, and used splice junctions (junction expression) in a variety of human tissues. This can give a rapid first indication of expressed transcripts in the tissue of interest. Even better is to also obtain RNA-seq data from samples with a wild type versus mutant status for the gene of interest in order to compare expressed transcript isoforms in these samples, as illustrated in Fig. 1. In an ideal world, this isoform expression analysis is based on long-read RNA-seq data generated on platforms such as PacBio or Nanopore sequencing. On such platforms, entire transcripts can be sequenced, allowing both accurate quantification of known transcripts as well as the discovery of *de novo* transcripts. If long-read RNA-seq is not possible, isoform expression analysis using short-read RNA-seq data is still an excellent alternative, which will allow to obtain similar performance in annotating the majority of mutations [15, 23]. The short- or long-read RNA-seq alignments (BAM files) can be visualized in IGV [27] to analyze expressed isoforms manually (Fig. 1). Furthermore, a number of computational tools are available to perform RNA-seq-based isoform quantification. For instance, tools such as IsoQuant, IsoTools, and FLAIR allow to reliably quantify known transcripts in long-read RNA-seq data, whereas Bambu, IsoQuant, and FLAIR are suitable for *de novo* isoform analysis in such data [28–32]. For short-read RNA-seq data, methodologies like Salmon, kallisto, RSEM, and Cufflinks can be applied for isoform expression analysis [33–37]. Whereas such bioinformatic tools are an excellent aid in isoform expression analysis, their accuracy is not 100% [28, 33]. Combining consistent results from different tools and/or manual verification of results of high interest in IGV is recommended to ensure correct isoform information to use as a basis for variant annotation. Finally, it is worth mentioning that researchers do not always have to generate the required RNA-seq data themselves. Indeed, such data are sometimes already available in public data portals such as the Genomics Data Commons (for cancer genomics data), from where they can be downloaded after completing a data access request in dbGAP. Also, in the field of genetic diagnosis of rare diseases, the added value of considering expressed RNA-transcripts when annotating mutations has been recognized. In this context, a transcript-level annotation metric known as the “proportion-expressed across transcripts” (or shortly “pext”) has been developed, which quantifies isoform expression for variants using GTEx expression data [25].

After obtaining information on the expressed transcripts, the next step is to annotate identified mutations in respect to these transcripts. Manual verification of the main expressed isoform in IGV, combined with manual variant annotation against this isoform gives the highest accuracy and is feasible when small numbers of variants need to be analyzed. For larger numbers of variants, automated methods are desirable. Different automated variant annotation tools are available, such as SnpEff [38], Variant Effect Predictor (VEP) [39], Variant Reporter (VR; a commercial software), and ANNOVAR [40]. Ideally, when using these tools, the user first selects the repertoire of transcripts to be used in annotation, based on what is found to be expressed in the sample or the corresponding healthy tissue. This avoids erroneous annotation in respect to irrelevant transcripts. In this regard, we showed that combining Salmon to analyze the main expressed isoforms in melanoma tumor samples in combination with VEP to annotate melanoma mutations to the expressed transcripts resulted in automated Salmon/VEP mutation annotation with 90% (45/50) accuracy. The remaining mutation annotation problems occurred because Salmon did not identify the correct main expressed transcript (3/50), because VEP could not annotate the mutation (1/50) or because the main expressed transcript was not in Ensembl (*de novo* transcript) (1/50) [15]. These problems may be resolved by combining results from different transcript quantification and variant annotation tools.

## Remaining challenges in coding/noncoding mutation annotation

**Figure 2.**
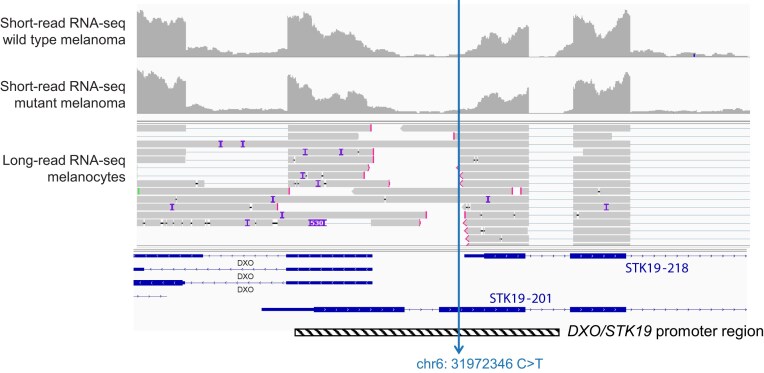
Challenges in annotating STK19 mutations. Short-read RNA-seq data from melanoma tumors were mapped against hg38 in IGV. A representative RNA-seq read distribution plot of a wild type and a mutant melanoma tumor is shown in the upper part of the panel. The genomic location of the *STK19 hotspot* mutation is indicated, as well as transcripts STK-201 and STK-218. The shown RNA-seq BAM files of melanoma tumors were downloaded from NCI Genomics Data Commons after having obtained dbGAP Authorized Access to dataset phs003014 (NCI’s Datasets for General Research Use, study accession phs000178.v11.p8—TCGA). In the middle, long-read RNA-seq data from Mel-ST melanocytes are shown. These data were described in [15] and are available for download at NCBI SRA (BioProject accession number: PRJNA1196620) and GEO (accession number: GSE290597).

Nevertheless, even when annotating in respect to expressed transcripts, certain challenges in mutation annotation remain. In some cases, multiple transcripts are expressed, for which a variant can have a conflicting coding or noncoding annotation. An excellent example to illustrate this concept are the mutations in the winged helix repair factor 1 (*WHR1*; better known as *STK19*) that are detected in 5% of melanoma tumors [41]. This gene displays a chr6:31972346 C > T variant, which was initially described as a p.Asp89Asn missense hotspot mutation [41, 42], corresponding to the annotation in respect to reference transcript *STK19-201* in Fig. 2. Yin *et al.* performed experiments in which cDNA corresponding to *STK19-201* was overexpressed in melanocytes. In this experimental setting, the STK19 p.Asp89Asn mutation behaved as gain-of-function missense substitution that enhances NRAS phosphorylation, melanocyte transformation, and NRAS-driven melanoma development in mice [42]. Interestingly, Rodríguez-Martínez *et al.* carefully analyzed expressed STK19 transcripts in different cell lines, including melanoma cell lines, and concluded that the expressed STK19 mRNA and protein correspond to a shorter isoform (transcript *STK19-218* in Fig. 2), in respect to which the mutation is noncoding. Furthermore, they showed that the chr6:31972346 C > T variant does not affect STK19 mRNA or protein expression levels. However, the data shown by Rodríguez-Martínez *et al.* do indicate a tendency for elevated STK19 mRNA expression in mutated versus wild-type melanoma tumors, and the data for the eight mutated patients that could be analyzed likely limit the statistical power to reach significance. Finally, they showed that STK19 is a nuclear protein that does not appear to affect NRAS signaling [43]. The findings of these two groups are thus in sharp contrast. Yin *et al.* reacted that Rodríguez-Martínez *et al.* are correct that *STK19-201* in respect to which the mutation is missense is not the main expressed isoform. However, they argue that there is also some RNA expression of *STK19-201*, and mass spectrometry peptides supporting expression of this protein isoform, and that the mutation may thus not be entirely noncoding as Rodríguez-Martínez *et al.* claim [44]. Indeed, as we show in Fig. 2, long-read Nanopore sequencing data from Mel-ST primary immortalized melanocytes support expression of different STK19 isoforms. Therefore, accurate annotation of this STK19 variant remains difficult, and it may have a mixed coding/noncoding annotation. Regarding the functionality of the mutation, Rodríguez-Martínez *et al.* concluded that the mutations do not affect STK19 expression levels, and hence inferred that these mutations are not functional. However, as also shown in Fig. 2, the mutations target a region that has been annotated as a shared promoter region for both the *STK19* and the *DXO* genes running in opposite directions. Rodríguez-Martínez *et al.* also show in their paper that, similar to what they see for STK19, DXO mRNA also tends to be more highly expressed in mutated patients compared to wild type, but again only eight mutated patients were available, limiting statistical power to reach significance. So, the annotation and functionality of these mutations, detected in 5% of melanoma patients, remain unclear at this point and would require further investigations in patient samples and accurate genetic models, such as isogenic melanoma cell lines in which the mutations are knocked-in by genetic engineering.

In addition to the problems highlighted above, results obtained with automated variant annotation tools can also vary significantly [45–47]. Yen *et al.* compared the performance of SnpEff, VEP, and VR across variant types using a curated set of 126 variants, following the Human Genome Variation Society (HGVS) guidelines. They highlighted challenges due to inconsistent coding and protein nomenclature across tools and databases like COSMIC and ClinVar. SnpEff and VEP demonstrated the best overall accuracy; VR performed well for SNVs but poorly for INDELs [45]. Chen *et al.* assessed concordance among ANNOVAR, SnpEff, and VEP using two-star clinically classified variants. SnpEff had the highest match for HGVS coding (0.988), while VEP excelled in HGVS protein annotation (0.977). However, significant discrepancies remain, especially for loss-of-function variants, indicating that this continues to be an open challenge.

Despite these challenges, accurate mutation annotation by integrating DNA- and RNA-seq data is a first step toward proper evaluation of the impact of a mutation. In the large majority of cases, such a strategy will result in accurate mutation annotation that can be used to design appropriate strategies to further experimentally test mutational impact as we explain in the next section.

## Utilizing accurate mutation annotation to evaluate mutational impact

Experimental approaches to analyze noncoding mutations are different from those that are relevant for synonymous and missense mutations [5, 48]. For instance, overexpressing a cDNA to model a synonymous or missense mutation in cells will only be relevant when the corresponding cDNA is actually expressed in the tissue where the mutation was initially identified [49]. In cases where another transcript is expressed, in respect to which the mutation is a noncoding promoter mutation, a (dual) luciferase reporter assay strategy testing transcriptional activity of the wild type versus mutated promoter is, for instance, more appropriate [8]. Also, in situations where a mutation affects splicing, cDNA overexpression is not appropriate, and a comparison of wild type versus mutant minigene reporters, containing both intronic and exonic sequences, may be a better alternative to assess the mutational impact on splicing [20, 50]. Sometimes, the exact nature of a mutation may be unclear when designing an experimental strategy, or a mutation may have a dual coding/noncoding annotation because different transcripts are expressed, as illustrated by the example of the *WNK1/STK19* mutations above. In such cases, generating isogenic models in which the mutation is knocked-in at the endogenous locus, for instance using CRISPR–Cas9 technology, is more appropriate, as no *a priori* knowledge of whether the mutation is coding or noncoding is required [15, 51]. The coding/noncoding nature of the mutations and their impact on gene expression and regulation can then be investigated in the generated models using techniques such as RNA-seq. Whereas such a CRISPR–Cas9 knock-in approach also avoids artifacts from supraphysiological expression levels and promoter sequences in a non-native environment, which can occur when using cDNA expression and promoter luciferase reporter assays respectively, it is much lower in throughput and requires substantially greater time and resource investment compared with cDNA expression and promoter reporter assays. Next-generation genome editing strategies such as base editing and prime editing have emerged as powerful alternatives for CRISPR–Cas9 that can be more cost and labor efficient. Furthermore, these more recent methods eliminate the need for double-strand breaks, thereby minimizing unintended genomic alterations. Base editing enables the precise conversion of one nucleotide to another without disrupting the DNA backbone, reducing reliance on error-prone repair pathways [52]. Prime editing further enhances precision by enabling targeted insertions, deletions, and modifications without requiring donor templates or introducing double-strand breaks [53]. These innovations mark a significant leap forward in genome editing applicability in research and allow to evaluate mutational impact in physiologically relevant experiments leaving native regulatory mechanisms intact. This also allows to provide relevant data to properly train machine-learning-based artificial intelligence (AI) tools to accurately predict mutational impact. Indeed, given the recent developments in the AI field, it is to be expected that clinical geneticists will in the future be assisted by powerful AI tools to evaluate the impact of identified mutations in clinical samples.

## General conclusion

**Figure 3.**
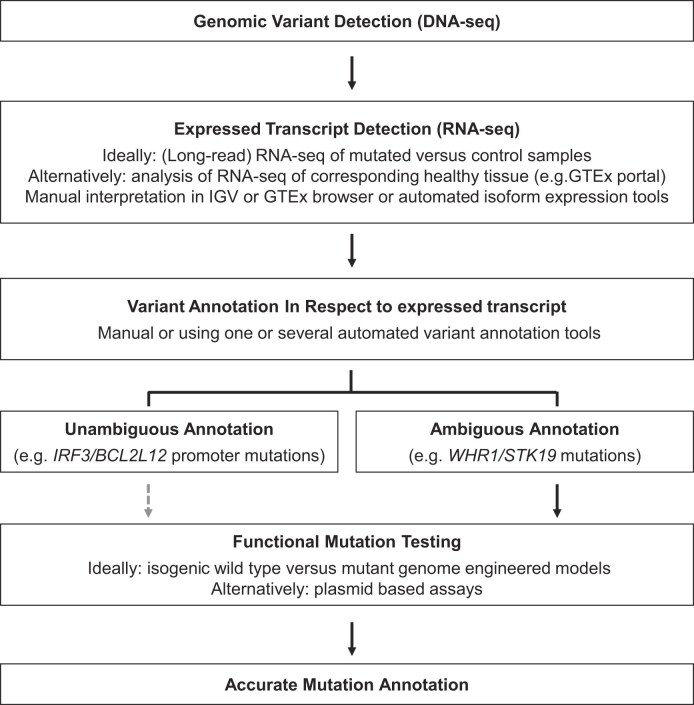
Recommended approach to avoid mutation misannotation. Flow chart summarizing our recommended approach to avoid misannotation of genetic variants. The gray interrupted arrow between “Unambiguous Annotation” and “Functional Testing” indicates that functional testing is optional to obtain accurate annotation when there is unambiguous annotation based on expressed transcripts. Such a testing can however still provide additional information of the impact of the mutation on gene expression and gene function.

In this perspective, we aim to illustrate the importance of integrating DNA- and RNA-seq data when annotating genetic variants. By annotating detected DNA mutations in respect to the expressed transcripts in the corresponding tissue or disease sample as detected by RNA-seq, major annotation errors that can arise from annotating relative to a nonexpressed reference transcript can be avoided. We propose possible solutions to improve the accuracy of variant annotation, and our recommended approach is summarized in Fig. 3. Ideally, RNA-seq results from samples that are wild type versus mutant for the variant under study are compared, as this can also reveal relevant changes in isoform expression, such as the ones driven by splice site mutations. Long-read RNA-seq-based methodologies are the best available technology to perform isoform expression analyses. However, these methodologies are not yet so widespread, and short-read RNA-seq data, as available for many patient samples in public data portals, offer an excellent alternative that often allows accurate mutation annotation. Nevertheless, even when evaluating expressed transcripts, annotation of certain mutations remains challenging, and functional testing in precisely gene-edited isogenic models is required to understand the effect of such mutations.

## Data Availability

No new data were generated or analyzed in support of this research.
